# Non-paroxysmal junctional tachycardia complicating disseminated intravascular coagulation during massive surgical hemorrhage: A case report

**DOI:** 10.1097/MD.0000000000037621

**Published:** 2024-04-05

**Authors:** Suyoun Chun, Sung Mee Jung

**Affiliations:** Department of Anesthesiology and Pain Medicine, Yeungnam University College of Medicine, Daegu, Republic of Korea.

**Keywords:** catecholamine, disseminated intravascular coagulation, hemorrhage, junctional tachycardia, systemic hypotension

## Abstract

**Rationale:**

Non-paroxysmal junctional tachycardia (NPJT) is a self-limiting supraventricular tachycardia associated with primary heart disease, cardiac surgery, digitalis toxicity, and metabolic or electrolyte imbalances. However, NPJT caused enhanced normal automaticity even in the absence of structural heart disease can be fatal if not managed properly.

**Patient concerns:**

A 74-year-old hypertensive female patient was scheduled for transureteroureterostomy and right ureteroneocystostomy under general anesthesia.

**Diagnosis:**

The patient developed NPJT without visible P wave and severe hypotension due to adrenergic stimulation in response to massive hemorrhage during surgery.

**Interventions:**

NPJT with hypotension was initially converted to sinus rhythm with normotension with administration of adenosine and esmolol. However uncontrolled surgical hemorrhage and administration of large dose of vasopressors eventually perpetuated NPJT refractory to antiarrhythmic drugs.

**Outcomes:**

Despite intravenous fluid resuscitation and massive transfusion, the patient was deteriorated hemodynamically due to uncontrolled bleeding and persistent NPJT, which resulted in hypovolemic shock and fatal disseminated intravascular coagulation (DIC).

**Lessons:**

NPJT can occur by enhanced automaticity due to increased catecholamine during severe surgical hemorrhage. Although NPJT is generally self-limiting, it can be refractory to antiarrhythmic agents and accelerate hypotension if the surgical bleeding is uncontrolled. Therefore, aggressive management of the primary pathologic condition is crucial for the management of NPJT and hemodynamic collapse even in the absence of structural heart disease.

## 1. Introduction

Non-reentrant junctional tachycardia originating from the atrioventricular (AV) junctional area encompassing the AV node and His bundle can be described based on etiology (primary and secondary) or timing (paroxysmal and non-paroxysmal). In contrast to the more rapid paroxysmal type, non-paroxysmal junctional tachycardia (NPJT) is characterized by gradual onset and termination of narrow QRS tachycardia between 70 and 130 bpm.^[1]^ Although its pathogenesis remains unclear, the mechanism of NPJT has been suggested to involve enhanced, abnormal automaticity or triggered activity.^[2]^

NPJT is a self-limiting supraventricular tachycardia usually associated with primary structural heart diseases, such as acute myocardial infarction, myocarditis, or cardiac surgery, or secondary to digitalis toxicity and metabolic or electrolyte imbalances including hypokalemia.^[3,4]^ However, acute hemorrhage-induced NPJT refractory to antiarrhythmic agents has not been previously described in adult patients with a structurally normal heart. Here, we report the case of an elderly hypertensive patient who developed NPJT due to adrenergic stimulation in response to a massive hemorrhage during urologic surgery, which resulted in hypovolemic shock and fatal disseminated intravascular coagulation (DIC).

## 2. Case report

**Figure 1.**
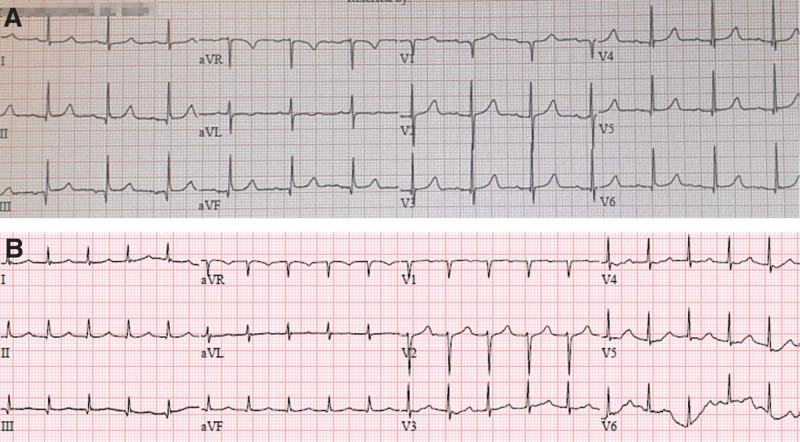
(A) The 12-lead electrocardiogram revealed NSR (80 bpm) before surgery and (B) NPJT (120 bpm) after surgery. NPJT = non-paroxysmal junctional tachycardia, NSR = normal sinus rhythm.

This case study was approved by institutional review board and informed consent was obtained from the legal guardian. A 74-year-old female (height: 153 cm; weight: 51 kg) with bilateral ureterovesical junction obstruction and hydronephrosis was scheduled for elective transureteroureterostomy and right ureteroneocystostomy under general anesthesia. Her medical history included dyslipidemia and hypertension with the use of calcium channel antagonist and beta blocker. She also had a history of undergoing 2 surgeries. The first was low anterior resection and hysterectomy after neoadjuvant radiotherapy for rectosigmoid cancer with uterine invasion 23 years prior. The second was an abdominoperineal resection (Mile’s operation) and small bowel resection for recurrent rectal cancer 8 months prior. At that time, the surgery was complicated by bilateral ureteral injury due to severe pelvic adhesions and continuous hemorrhage. Postoperatively, her clinical course was complicated by coagulopathy-induced hemorrhage requiring a large number of blood components and sepsis with the transient development of atrial fibrillation. Despite bilateral ureteral percutaneous nephrostomy, she suffered from ineffective urine drainage requiring hemodialysis and eventually chronic kidney disease (CKD) with metabolic acidosis. Preoperative electrocardiography (ECG) revealed normal sinus rhythm (NSR) with a heart rate of 80 beats/min (bpm) (Fig. 1). Her pulmonary function test results were normal, despite bronchitis with an emphysematous pattern and atherosclerotic cardiovascular changes on chest radiography. The preoperative laboratory examination revealed anemia (8.7 g/dL), hyperglycemia (136 mg/dL), hyperkalemia (5.4 mmol/L), and increased BUN and creatinine levels (64.3 mg/dL and 4.28 mg/dL, respectively) with a reduced estimated glomerular filtration ratio (estimated glomerular filtration ratio; 10.1 mL/min/1.73 m^2^). On the morning of the surgery, her Hb was increased to 11.1 g/dL, and her serum potassium and glucose levels were 5.1 mmol/L and 82 mg/dL, respectively.

On arrival in the operating room without premedication, standard monitoring revealed an NSR (68 bpm), noninvasive blood pressure of 180/75 mm Hg, and SpO_2_ of 98%. General anesthesia was induced with propofol (50 mg), and endotracheal intubation was facilitated after the administration of cisatracurium 10 mg. Anesthesia was maintained with 4% to 5% desflurane to achieve a bispectral index range of 40 to 60 and continuous infusion of remifentanil and cisatracurium during surgery. Mechanical ventilation with a mixture of oxygen and air (FiO_2_ 50%) was controlled to achieve normocapnia. After anesthesia induction, cannulation of the radial artery and peripheral vein was established for hemodynamic monitoring and administration of fluids and medications, respectively. Forced air warming was used to maintain normothermia.

**Figure 2.**
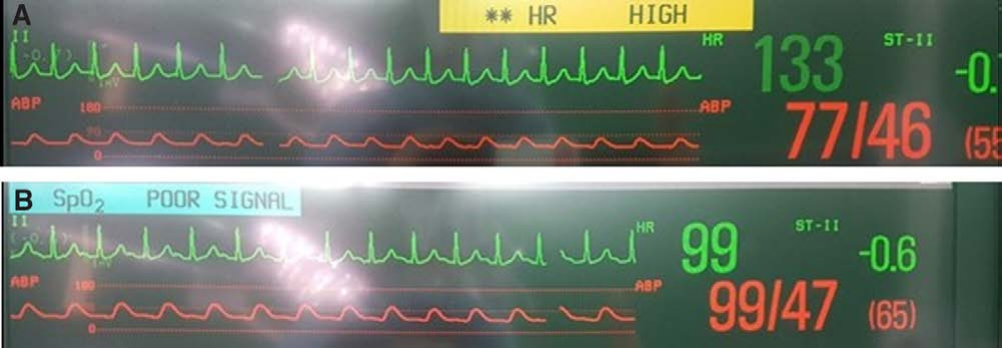
(A) The lead II rhythm strip showed a regular narrow QRS complex rhythm (rate 133 bpm) without premature atrial or ventricular fusion, consistent with NPJT The P waves were invisible, which are buried in the QRS complex due to simultaneous activation of the atria and ventricles by the junctional (nodal) pacemaker. (B) Intravenous injections of adenosine 6 mg converted to sinus rhythm. NPJT = non-paroxysmal junctional tachycardia.

A systolic blood pressure (SBP) of 140 to 160 mm Hg and a heart rate of 60 to 70 bpm were maintained with adequate urine output after surgical incision (Table 1). Subsequently, her heart rate gradually increased to 90 to 100 bpm with an SBP of 110 to 120 mm Hg 2.5 hours later. Arterial blood gas analysis showed metabolic acidosis (pH 7.238, PaCO_2_ 38.1 mm Hg, base excess −11.1 mmol/L, lactate 1.4 mmol/L) with a normal range of hemoglobin and electrolyte levels. We performed a preload challenge using crystalloids to preserve circulatory volume. Then, 3 hours and 10 minutes after commencing the operation, approximately 300 mL of blood abruptly flowed out of the surgical site. At the same time, narrow regular QRS complex tachycardia without a clearly visible P wave at a rate of 130 to 140 bpm was accompanied by a significant reduction in SBP from 110 to 120 mm Hg to 70 to 80 mm Hg 15 minutes later (Fig. 2). Her SpO_2,_ pressure of end-tidal CO_2_, peak airway pressure, and body temperature remained unchanged.

**Table 1:** Hemodynamic variables and laboratory test.

Elapsed time	0 h	2 h 30 min	3 h 10 min	3 h 25 min	4 h 30 min	5 h 20 min	7 h 30 min
Event	Skin incision	Increase in heart rate	Acute blood loss	Onset of NPJT	Impaired hemostasis	coagulopathy-induced bleeding	Surgical closure
ECG	SR	SR	SR	NPJT & SR	NPJT	NPJT	NPJT
Management	Nicardipine for hypertension	Fluid loading	Fluid loading	Adenosine esmolol NE fluid loading	NE, E Vasopressin fluid loading transfusion	NE, E Vasopressin Massive transfusion	NE, E Vasopressin Massive transfusion
SBP (mm Hg)	140–160	110–120	70–100	60–70	50–70	70–80	80–90
Heart rate (bpm)	60–70	90–100	110–120	100–140	100–120	100–120	100–120
pH		7.238	7.222	7.167	7.169	7.216	7.217
PaCO_2_ (mm Hg)		38.1	41.7	53.4	47.4	49.5	44.1
PaO_2_ (mm Hg)		127	136	116	79	113	107
BE (mmol/L)		−11.1	−10.6	−9.3	−11.3	−7.7	−9.9
Hb (g/dL)		10.7	10.9	8.5	7.0	9.3	10.8
K (mmol/L)		4.6	4.7	4.6	4.2	4.4	4.5
Glucose (mg/dL)		108	114	81	69	72	91
Lactate (mmol/L)		1.4	2.2	3.3	4.4	5.3	6.0
Temperature (°C)		36.7	37.0	36.8	36.2	35.6	35.1

BE = base excess, E = epinephrine, ECG = electrocardiography, N = norepinephrine, NPJT = non-paroxysmal junctional tachycardia, SBP = systolic blood pressure, SR = sinus rhythm.

**Figure 3.**
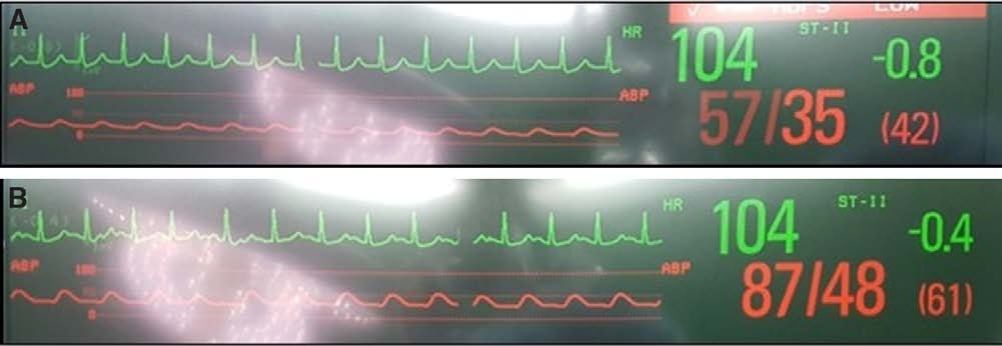
NPJT (A) resulted in much lower blood pressure compared to sinus tachycardia (B), even at the same heart rate. NPJT = non-paroxysmal junctional tachycardia.

The vagal maneuver was ineffective. The administration of ephedrine and phenylephrine to increase blood pressure also failed. Suspicious of junctional tachycardia, an intravenous flush of adenosine 6 mg immediately converted to a sinus rhythm at a heart rate of 90 to 100 bpm with an SBP of 100 to 110 mm Hg. However, 10 minutes later, junctional tachycardia with systemic hypotension reappeared. Arterial blood gas analysis showed progressive metabolic acidosis (pH 7.222, base excess −10.6 mmol/L, lactate 2.2 mmol/L) but hemoglobin and electrolyte levels remained unchanged. Repeated administration of adenosine and esmolol returned the sinus rhythm with an elevated SBP. However, the sinus rhythm recovery period gradually shortened over approximately 1 hour, and eventually, junctional tachycardia persisted following the 5th recurrence. During the alteration of the cardiac rhythm, junctional tachycardia was consistently associated with a higher heart rate and lower blood pressure than sinus tachycardia (Fig. 3).

Despite the administration of vasopressors (norepinephrine, epinephrine, and vasopressin), aggressive fluid replacement, and transfusion of packed red blood cells through 2 newly secured intravenous routes, systemic hypotension persisted due to continuous bleeding from the surgical site since the initiation of junctional tachycardia. Moreover, 2 hours after the onset of junctional tachycardia, the amount of blood loss progressively increased, with a decrease of the SBP to 50 to 60 mm Hg. At this time, the surgeon was informed of the difficulty of achieving hemostasis in the surgical field. Extremely hypovolemic ventricles with well-preserved wall motions were observed on transesophageal echocardiography. Laboratory tests revealed progressive lactic acidosis (lactate 3.3 nmol/L). Coagulation profiles with thrombocytopenia (platelet count 25,000/μL), prolonged clotting time (prothrombin time 45.9 seconds with INR 3.98 and unmeasurable activated partial thromboplastin time), hyperfibrinogenemia (0.63 g/dL) and elevated D-dimer level (24.94 μg/mL) indicated DIC. Troponin I and CK-MB levels were normal. Her peak airway pressure gradually increased to 31 mm Hg with decreased oxygenation (PaO_2_ 76 mm Hg), and petechiae were observed on the edematous skin. Despite massive transfusion of blood components and administration of antifibrinolytics, generalized bleeding with severe hemodynamic instability persisted. Eventually, surgery lasting 8 hours was terminated with a heavy gauze retention suture without a definite bleeding focus identified at the surgical site. Intraoperative blood loss was estimated to be 8000 mL. The urine output was 450 mL for 3 hours before the onset of acute bleeding and could not be measured after that. The patient received 6200 mL of intravenous fluid, 25 units of packed red blood cells, 10 units of fresh frozen plasma, and 8 units of platelet concentrate during the operation.

On arrival in the intensive care unit without extubation for further management, the SBP was maintained between 90 and 100 mm Hg with junctional tachycardia (100–140 bpm) and a prolonged QT interval (QT_c_ 508 ms, Fig. 1). Blood and tracheal aspirate cultures obtained from the intensive care unit yielded negative results but blood seeping through the dressing and wound drain, rapidly filled with blood was sustained. Her hemodynamics progressively deteriorated despite resuscitation with vasopressors and fluid, massive transfusion of blood components, and DIC treatment. Resuscitation was discontinued 22 hours after the operation as further attempts were unlikely to be beneficial.

## 3. Discussion

Here, we report the occurrence of NPJT resulting from adrenergic stimulation in response to a massive hemorrhage in a patient during noncardiac surgery. In the early stages of hemorrhage, NPJT was converted to NSR by treatment with adenosine and beta blocker. However, despite aggressive fluid replacement and massive transfusion, uncontrolled hemorrhage and exogenous administration of catecholamine perpetuated NPJT with hemodynamic deterioration, which consequently resulted in hypovolemic shock combined with fatal DIC in the present case.

As a non-reentrant SVT, NPJT appears as a regular narrow QRS complex tachycardia with gradual onset and may have either AV dissociation or 1:1 ventriculoatrial conduction. In the present case, NPJT was diagnosed as narrow QRS complex tachycardia without visible P waves while the sinus rate gradually increased in response to surgical hemorrhage. With invisible P waves and a lack of a temporal relationship between P and the QRS complex, we could not clarify the presence of AV dissociation but could infer the pacemaker site of this rhythm. When an electrical impulse originates in the compact AV node, the distance that the impulse must travel up through the atria (retrograde) and down through the ventricles (anterograde) is almost the same.^[5]^ At this time, the P wave is buried or hidden by the QRS complex because the force of atrial depolarization is less than that of ventricular depolarization. The QRS duration and morphology similar to normally conducted beats indicate that the electrical impulse originating above the branching portion of the His bundle follows a normal conduction pathway through the ventricles. The rhythm was regular because the RR intervals were regular and equal in length, resulting from a 1:1 exit from a single ectopic focus. Due to its rarity in the adult population and variable presentation, the diagnosis of NPJT must be differentiated from other types of regular, narrow QRS complex tachycardia, such as atrial tachycardia, AV node reentrant tachycardia and AV reentrant tachycardia. Spontaneous initiation of tachycardia without premature atrial or ventricular depolarization can help distinguish NPJT from AV node reentrant tachycardia or AV reentrant tachycardia. No clearly distinguishable P waves can exclude atrial tachycardia.

NPJT occurs primarily in patients with structural heart disease associated with inflammation near the AV junction or secondary to autonomic or metabolic disturbances or digitalis toxicity. Our patient had no known structural heart disease or clinical signs suggestive of acute myocardial ischemia, such as changes in the ST segment, cardiac regional wall motion abnormalities, or elevated cardiac enzyme levels in the perioperative period. Her electrolyte (potassium) and glucose levels remained within acceptable ranges during an episode of NPJT. Metabolic acidosis due to CKD by itself cannot explain the intraoperative occurrence of NPJT, as it persisted for several months prior to surgery. The effects of general anesthetics on arrhythmogenesis can also be excluded since sinus rhythm was maintained until 4 hours after the induction of anesthesia.

The mechanism of acceleration beyond physiological automaticity (its usual rate of 35–36 bpm) in AV junctional discharge is thought to be enhanced, abnormal automaticity, or triggered activity.^[2]^ We had to ascertain the mechanism of NPJT based on the surface ECG findings and clinical course since an electrophysiological study could not be performed because of hemodynamic deterioration in the present case. Considering that NPJT without premature or fusion complexes spontaneously appeared at a faster rate than sinus rhythm during a gradual increase in sinus rhythm, enhanced automaticity by a reflex increase in adrenergic drive may play an important role. The response to pharmacological management also provided clues regarding the mechanism of NPJT in our patient. Under catecholamine stimulation, adenosine suppresses AV nodal conduction by directly activating adenosine-sensitive potassium channels, while it suppresses AV automaticity by indirectly antagonizing beta-adrenergic mediated increase in pacemaker current (I_f_) and calcium current (I_ca_).^[6–8]^ The conversion of NPJT to sinus rhythm without AV nodal block by adenosine and beta blocker is consistent with the catecholamine-enhanced automatic mechanism in the present case.

Sinus and junctional pacemakers show similar directional changes in response to catecholamine infusion.^[9]^ Adrenergic stimulation increases the pace-making rate via the norepinephrine-mediated increase in the activity of the stimulatory G-protein, which increases cAMP production and the open probability of the hyperpolarization-activated cyclic nucleotide-gated channel for pace-making I_f_ current. Consequently, this increases the slope of phase 4 depolarization and shifts the threshold to a more negative level in the AV junction, even more than the sinoatrial (SA) node.^[10,11]^ In the present case, sinus and junctional tachycardia occurred at a relatively similar rate range, suggesting that both sinus and junctional pacemakers were influenced and mathematically related.^[3,12]^ Thus, if catecholamine-enhanced automaticity plays a role, junctional automaticity would increase during the more rapid phase of the sinus rhythm and supersede the sinus rhythm only after the pacemaker activity of the SA node is suppressed. Considering the history of transient symptomatic atrial fibrillation during the last postoperative period and underlying conditions (hypertension and CKD with metabolic acidosis),^[13,14]^ the patient probably had potential SA node dysfunction. In addition, massive bleeding can reduce blood flow to the SA node and thus mask SA node dysfunction. Thus, adrenergic stimulation in response to hemorrhagic shock may have shifted the dominant pacemaker site from the SA node to the AV junction^[15]^ in the present case.

The clinical significance of NPJT is generally related to the severity of the underlying heart disease rather than to the rhythm itself.^[4]^ NPJT regresses spontaneously as the underlying pathophysiological condition is corrected or may be successfully treated with procainamide, potassium salts, or propranolol.^[1,3]^ However, NPJT can be fatal because of hypotension and hemodynamic collapse if the primary conditions are uncontrolled. In the present case, NPJT was initially converted to NSR by adenosine and beta blocker but reappeared and persisted with a sustained increase in circulating epinephrine and norepinephrine in response to hypovolemic shock and the exogenous administration of catecholamine. In addition, because of the reduction in left ventricular end-diastolic volume due to the loss of atrioventricular synchrony, NPJT results in much lower blood pressure than sinus rhythm, even at the same heart rate,^[15]^ which may lead to the further aggravation of hemodynamic instability and DIC. Therefore, the primary pathophysiological condition must be treated before establishing a management plan for junctional tachycardia especially in hemodynamically unstable patients.

There are some limitations in this case study. First, we could not perform electrophysiological study to differentiate NPJT from other narrow QRS supraventricular tachycardia because of hemodynamic deterioration in the present case. We should determine and treat arrhythmia and hemodynamic collapse depending on the surface ECG and initial response to antiarrhythmic agents. Second, because the arrhythmia occurred simultaneously with apparent hemorrhage, we initially could not infer a relationship between the 2 and attempted to treat each separately. Vigilant monitoring of hemodynamic parameter and surgical field is necessary to determine the mechanism of arrhythmia and primary pathologic condition in surgical patients.

In summary, NPJT originating from the AV node can occur when the junctional rate is greater than the sinus rate, owing to catecholamine-enhanced automaticity in response to acute severe hemorrhage in patients without structural heart disease. Interestingly, treatment with adenosine and a beta blocker or an increase in blood pressure with volume replacement can decrease junctional automaticity and recover pace-making automaticity in SA nodes. Therefore, aggressive treatment of the primary pathophysiological conditions as well as junctional tachycardia is required to maintain hemodynamic stability when NPJT occurs by catecholamine-enhanced automaticity.

## Author contributions

**Conceptualization:** Sung Mee Jung.

**Data curation:** Suyoun Chun, Sung Mee Jung.

**Formal analysis:** Suyoun Chun, Sung Mee Jung.

**Investigation:** Suyoun Chun, Sung Mee Jung.

**Supervision:** Sung Mee Jung.

**Visualization:** Sung Mee Jung.

**Writing – original draft:** Suyoun Chun.

**Writing – review & editing:** Suyoun Chun, Sung Mee Jung.
